# Smallpox vaccination campaigns resulted in age-associated population cross-immunity against monkeypox virus

**DOI:** 10.1099/jgv.0.001999

**Published:** 2024-06-11

**Authors:** Kieran Dee, Maria Manali, Laura A. Bissett, Jordan Bone, Callum Magill, Chris Davis, Brian J. Willett, Pablo R. Murcia

**Affiliations:** 1 MRC-University of Glasgow Centre for Virus Research, Glasgow, G61 1QH, UK

**Keywords:** cross-immunity, Mpox, smallpox vaccination, antibody-mediated immunity

## Abstract

Increased human-to-human transmission of monkeypox virus (MPXV) is cause for concern, and antibodies directed against vaccinia virus (VACV) are known to confer cross-protection against Mpox. We used 430 serum samples derived from the Scottish patient population to investigate antibody-mediated cross-neutralization against MPXV. By combining electrochemiluminescence immunoassays with live-virus neutralization assays, we show that people born when smallpox vaccination was routinely offered in the United Kingdom have increased levels of antibodies that cross-neutralize MPXV. Our results suggest that age is a risk factor of Mpox infection, and people born after 1971 are at higher risk of infection upon exposure.

## Introduction

Mpox is a zoonotic disease caused by monkeypox virus (MPXV), a member of the *Orthopoxvirus* genus of viruses [1]. This group consists of viruses with wide-ranging tropism and virulence. Some orthopoxviruses that infect animals are associated with zoonotic infections, like MPXV or Cowpox virus [2], while others can infect only humans, such as Variola virus (VARV) the causative agent of smallpox. Mpox used to be reported mainly in countries in Central and Western Africa, but as of August 2023 it has been reported in 113 countries, with 89 308 laboratory-confirmed cases and 152 deaths [3]. This unprecedented spread of the virus led to the declaration of a Public Health emergency by the World Health Organization (WHO).

The structural proteins of orthopoxviruses exhibit high levels of sequence similarity. This was exploited during the smallpox vaccination campaign as people were inoculated with vaccinia virus (VACV), which did not cause disease but induced immunity against VARV. The smallpox vaccination campaign ended in the United Kingdom (UK) in 1971 [4] and the last naturally occurring case was in 1977 in Somalia [5]. Immunization with virus strains based on the replication-deficient Modified Vaccinia Ankara-Bavarian Nordic (MVA-BN) vaccine confers long-lasting humoral immunity [6], with similar litres of antibodies seen in both young and old people who would have been vaccinated or exposed to VARV [7]. Vaccination with MVA-BN also confers protection against infection with MPXV in both challenge and observational retrospective cohort studies [8,9]. Neutralizing antibodies are key for protection since the development of an immunoglobulin G (IgG) response is correlated with lower viral load and reduced disease severity [10]. Given the timeline of the smallpox vaccination campaign in the UK, the cross-protection induced by MVA-BN, and the long lifespan of vaccine-induced antibodies, we hypothesized that individuals vaccinated against smallpox when vaccination campaigns were still active might still have detectable antibodies against VACV that would cross-neutralize MPXV and thus might offer protection against Mpox.

## Methods

### Serum samples

Four-hundred-and-thirty serum samples were selected from a biobank of residual biochemistry specimens obtained from adult patients from primary and secondary healthcare settings. Serum samples were collected between March 2020 and September 2022. Associated metadata included date of sample collection, sex, and date of birth. Samples were divided into eleven age groups. Using Cochran’s formula, we determined that 20 samples per group would give 95 % confidence to detect statistical differences between age groups.

### IgG quantification

IgG antibodies against VACV (A27L; D8L; L1R; A33R; B5R) and MPXV (A29L; E8L; M1R; A35R; B6R) were measured using the Meso Scale Discovery (MSD) V-PLEX Orthopoxvirus Panel 1 (IgG) kit (Catalogue # K156882U). MSD electrochemiluminescence assays were performed according to the manufacturer’s instructions. Briefly, 96-well plates were blocked at room temperature for at least 30 min. Plates were washed; samples diluted (1 : 500) and added to the plates along with reference standards and controls. Plates were incubated for 2 h and further washed. SULFO-TAG detection antibody was added, and plates were incubated for 1 h. After incubation, plates were washed and read using a MESO Sector S 600 plate reader. Electrochemiluminescence data were processed using Methodological Mind software and analysed using MSD Discovery Workbench (version 4.0). Results were normalized to standard(s) provided with the MSD kit and expressed as MSD arbitrary units per millilitre (AU ml^−1^). Electroluminescence data for all antigens are provided in Table S1, available in the online version of this article.

### Viruses and cells

MPXV-CVR-S1 (accession number ON808413.1) was used. VACV (Western Reserve strain) was obtained from BEI Resources (catalogue number NR55). Virus stocks were grown in Vero 6F5 cells. Virus was harvested when cytopathic effect was observed. Virus stocks were prepared from cell lysates. Cell culture media consisted of Dulbecco’s minimum essential media (DMEM) glutamax with 2 % fetal bovine serum (FBS).

### Virus neutralization assays

In total, 2×10^5^ cells ml^−1^ Vero 6F5 cells were seeded ~24 h before infection. For the initial screen sera were diluted 1 : 25 in DMEM with 2 %FBS and 1 % non-essential amino acids (NEAA). Then 60 µl of infectious medium containing 3×10^4^ plaque forming units (PFU) of either VACV or MPXV was added to 60 µl of each serum dilution (1 : 25) in triplicate. The mixtures were incubated at 37 °C for 1 h and 100 µl was added to the cells. The input control consisted of 60 µl of virus being added to 60 µl of DMEM 2 %FBS/1 %NEAA. A monoclonal antibody targeting MPXV M1R (Clone: MPXV-26, Leinco Technologies, Inc.) was used as a positive neutralization control. For VACV and MPXV assays, the plates were incubated at 37 °C 5 %CO_2_ for 10 and 16 h, respectively and then fixed in 8 % formaldehyde. Cells were permeabilized with 1 % Triton-X for 10 min. Virus positive cells were detected using a polyclonal antibody raised against VACV (Invitrogen: PA1-7258) at a dilution of 1 : 1000 and then a secondary antibody (goat anti-rabbit IgG Alexa Flour 488, A11034) also at 1 : 1000. Positive cells were counted using a Nexcelom Celigo imaging cytometer. For each plate, the mean total count for the input control was calculated. The total virus-positive cell count for each sample was then expressed as a percentage of the mean input count. These values were subtracted from 100, to give a percentage neutralization value. Neutralization data for each virus is provided in Table S1.

## Results

### Presence of antibodies binding to VACV and MPXV epitopes is associated with year of birth

**Fig. 1.**
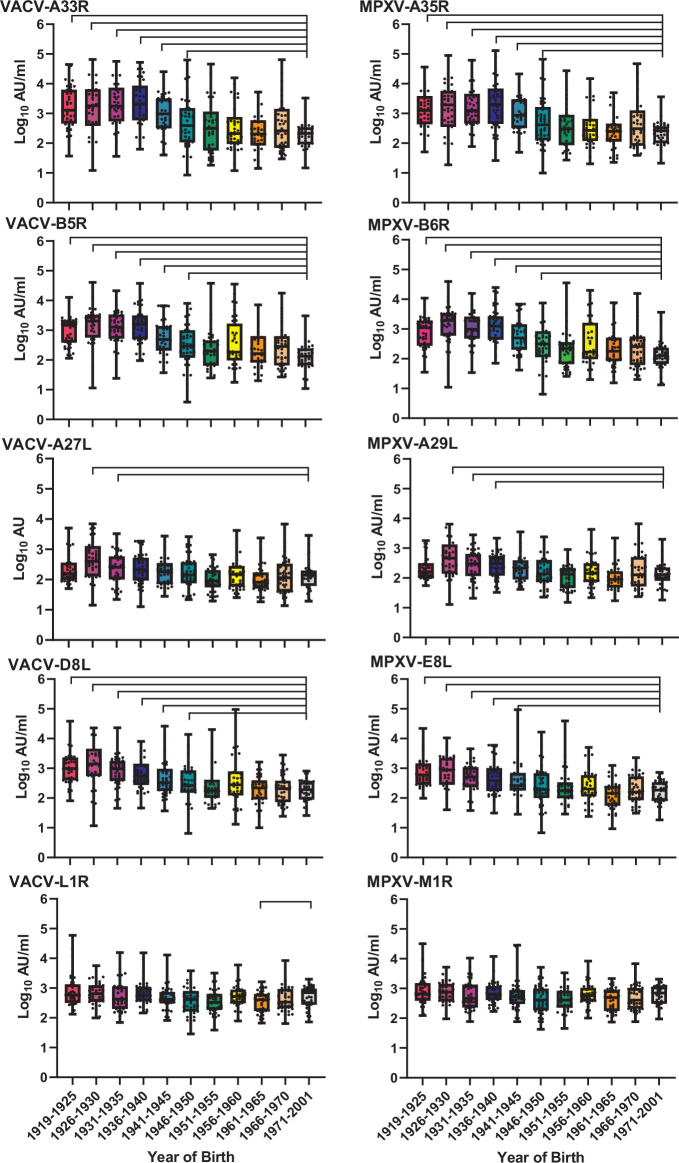
Levels of anti-VACV and anti-MPXV antibodies in sera from individuals born before and after the interruption of smallpox vaccination campaigns. In total, 430 sera samples were screened against the antigens shown. Antibody concentrations are shown in Meso Scale Discovery (MSD) log_10_ arbitrary units (AU). Box plots display the median and quartile values and whiskers show highest and lowest values for each group. Statistical significance of antibody levels between between the 1971–2001 and all the other age groups was tested using pairwise Wilcoxon test. *P* values<0.05 are displayed.

As vaccination against smallpox ended in the UK in 1971, we were interested to investigate the prevalence of antibodies present in people who were born before or after that year. We utilized the MSD immunogenicity assay to detect antibodies against VACV and MPXV from 430 serum samples. This assay utilized five homologous gene products of both VACV and MPXV: three of them (A27L; D8L; L1R -VACV and A29L; E8L; M1R -MPXV) were associated with the intracellular mature virus whilst the other two (A33R; B5R -VACV and A35R; B6R -MPXV) were associated with the extracellular envelope virus particle. Fig. 1 shows the relative levels of antibodies as measured by the MSD sandwich electrochemiluminescence immunoassay for all the samples tested. Age groups were stratified by year of birth, with the oldest group born between 1919–1925. The youngest group represents people born between 1971 and 2001. Because we were primarily interested in comparing antibody levels between serum samples pre- and post-widespread vaccination, samples from patients born between 1971–2001 were grouped together, whilst samples from other groups were divided in 5 year intervals. We observed a clear trend in the relative levels of antibodies with year of birth, with higher levels of antibodies detected in older patients. We found statistically significant differences in the levels of antibodies in sera from people born between 1971–2001 than those from older groups (Fig. 1). For example, for VACV-A33R and MPXV-A35R we found significantly higher levels of antibodies in sera from people born between 1919 and 1950 than for those born between 1971–2001 (*P* value<0.05, Fig. 1). We observed no significant difference between the youngest group and any other group for MPXV-M1R and borderline significant differences in levels of anti-VACV-L1R in the 1971–2001 group compared to the 1961–1965 group (*P* value 0.0471, Wilcoxon test, Fig. 1). Overall, trends in antibody levels were similar for both VACV and MPXV antigens and antibody levels strongly correlated between the homologous gene products of VACV and MPXV, implying strong cross-reactivity (Fig.S1).

### VACV and MPXV are significantly less neutralized by sera from patients born after 1971

**Fig. 2.**
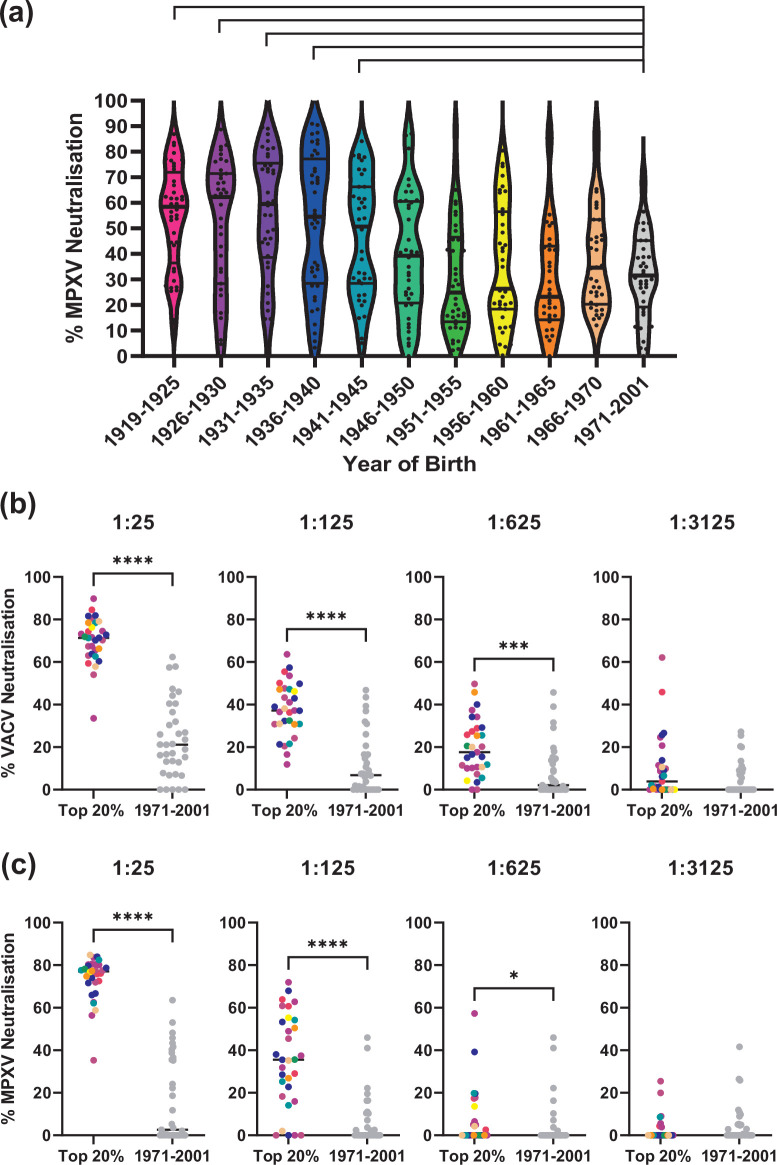
VACV and MPXV are significantly less neutralized by sera from patients born after 1971. (**a**) A total of 430 serum samples were tested for their capacity to neutralize MPXV. Data points represent percent MPXV neutralization for each serum sample tested. The thick black bar represents the median and the narrow bars represent the upper and lower quartiles. Statistical significance of neutralization values between the 1971–2001 and all the other age groups was tested using a Wilcoxon test and any comparison with a *p* value<0.05 is displayed. (**b**) VACV is more neutralized by sera taken from the top 20 % neutralizing samples. Serum samples with the highest neutralization values from the 430 sample screen were diluted and tested for their neutralizing capacity against VACV. These were compared to samples taken from the 1971–2001 age group. (**c**) MPXV is more neutralized by sera taken from the top 20 % neutralizing samples. Serum samples with the highest neutralization values from the 430 sample screen were diluted and tested for their neutralizing capacity against VACV. These were compared to samples taken from the 1971–2001 age group at the same dilutions. Significance levels between the groups were tested using a Wilcoxon test. *P<0.05. ***P<0.001, ****P<0.0001.

As antibody-mediated neutralization is a correlate of protection against infection with orthopoxviruses [10], we investigated if the increased levels of antibodies against either VACV or MPXV would translate to increased neutralization of live virus. To this end, we examined the neutralization ability of our serum samples against MPXV at a fixed dilution (1 : 25). Consistent with our result for MPXV antigen binding activity (Fig. 1) we observed a clear trend showing increased MPXV neutralization with increasing age. When compared to the 1971–2001 age group, we recorded significantly higher levels of neutralization of MPXV in the age groups 1919–1925 (*P* value: 8.92×10^−6^, Wilcoxon test), 1926–1930 (*P* value: 5.35×10^−4^, Wilcoxon test), 1931–1935 (*P* value: 1.92×10^−5^, Wilcoxon test), 1936–1940 (*P* value: 2.7×10^−4^, Wilcoxon test), and 1941–1945 (*P* value: 0.005, Wilcoxon test) (Fig. 2a). No significant differences were seen between the 1971–2001 age group and any age group born after 1945.

To characterize the cross-neutralization potency of anti-VACV antibodies in patients' sera, we repeated the neutralization assays against MPXV and VACV in a subset (*n*=31) of samples using multiple dilutions (1 : 125; 1 : 625; and 1 : 3125). Serum samples that exhibited the top 20 % neutralization levels against MPXV were compared with a subset of samples from the 1971–2001 group (*n*=35). Fig. 2 shows significant differences in neutralization against VACV and MPXV even at a 1 : 625 dilution, which is remarkable as our serum samples were collected between 2020 and 2022, approximately 50 years after routine vaccination against smallpox was halted in the UK. Moreover, samples with neutralization levels of 80 % or higher were derived from patients born between 1921–1969, and most of these samples (80.6 %) were from patients born between 1921–1950. Overall, these results show that antibody-mediated immunity against VACV is long-lasting and provides cross-neutralization against MPXV.

## Discussion

We investigated the presence of cross-neutralizing antibodies against MPXV in the Scottish patient population, which included people who would have been eligible to receive a smallpox vaccine before routine administration in the UK stopped in 1971. Since humoral immunity wanes over time, it is notable that serum from patients who may have either been vaccinated against smallpox or exposed to VARV decades ago not only have anti-VACV antibodies, but also that they retain high levels of neutralizing and cross-neutralizing activity. Our results are consistent with the detection of antiviral antibodies up to 75 years after smallpox vaccination [11] and agree with a previous study that found similar levels of anti-VACV antibodies in serum samples taken from the same patients 10 years apart. This study also found comparable antibody titres between people aged 30 and 100 years of age, which shows the remarkable longevity of anti-orthopoxvirus antibodies [7]. We observed a moderate correlation between virus neutralization and detection of serum antibodies, the strongest correlation being between neutralization and detection of anti-B6R antibodies (ρ:0.538, Spearman’s rank correlation, Fig. S2) suggesting that detection of antibodies against certain epitopes might be used as markers of protection. We also observed a strong correlation between antibody levels for homologous gene products of VACV and MPXV, with the strongest correlation seen between VACV-B5R and MPXV-B6R and VACV-L1R and MPXV-M1R (ρ:0.99, for both comparisons, Spearman’s rank correlation, Fig. S1). This agrees with previous studies that show strong cross-reactivity between MPXV and VACV [12].

A recent meta-analysis shows significant correlation between VACV-induced antibodies and vaccine effectiveness against mpox [13]. However, the authors emphasize that additional work is needed to validate such correlation. Interestingly, the youngest age groups with significantly higher levels of antibodies than the cohort that were born after smallpox vaccination ended in the UK were born before 1951 (Fig. 1, MPXV-A33R/B5R and VACV-A35R/B6R). However, the youngest age groups that had significantly higher neutralization levels against MPXV were born before 1946. This is most likely reflective of a dwindling of vaccination campaigns as smallpox cases began to become more and more sporadic. It is feasible to think that people born after 1950 would have been less likely to receive an immunization or be exposed to VARV than someone who would have been born earlier, when the threat of the smallpox was more tangible. Smallpox ceased to be endemic in the UK in the 1930s and mandatory vaccinations ended in 1948. There were outbreaks in Scotland in 1937, 1942 and 1950 [4]. The outbreak of 1950 sparked a widespread vaccination campaign, resulting in 300 000 people being vaccinated in Lanarkshire and Renfrewshire shortly thereafter [4]. In this context, it is interesting to note that the groups that had significantly higher antibody titres for A33R, A35R, B5R, B6R, and D8L were all born before this occurred, and that the groups born after this mass vaccination event do not show significantly greater levels of anti-orthopox antibodies compared to the 1971–2001 group.

Some serum samples in the 1971–2001 age group exhibited an intermediate level of neutralization against MPXV and VACV, which was higher than we expected. This is consistent with previous serology data [14], and has also been reported for T-cell mediated immunity where CD4^+^ and CD8^+^ T-cells showed reactivity to orthopox-derived peptide pools [15]. We speculate that this observation may be the result of exposure of those patients to other orthopoxviruses that circulate in animals, such as cowpox or Ectromelia virus.

The COVID-19 pandemic underscored the impact of severe infections in the elderly on national health services around the world. As people tend to live longer, our results provide important information on the beneficial and long-lasting effects of vaccination against orthopoxvurses if MPXV (or other orthopoxvirus) was to emerge.

## supplementary material

10.1099/jgv.0.001999 Uncited Fig. S1.

10.1099/jgv.0.001999 Uncited Table S1.

## Abbreviations

AU: arbitrary units
COVID-19: coronavirus disease
DMEM: Dulbecco's minimum essential media
FBS: fetal bovine serum
IgG: immunoglobulin G
ml: millilitre
MPXV: monkeypox virus
MSD: Meso Scale Discovery
MVA-BN: Modified Vaccinia Ankara-Bavarian Nordic
NEAA: non-essential amino acids
PFU: plaque forming units
UK: United Kingdom
VACV: vaccinia virus
VARV: variola virus
WHO: World Health Organization
