# Supplementary material for: Smallpox vaccination campaigns resulted in age-associated population cross-immunity against monkeypox virus
Source: J Gen Virol. 2024 Jun 11;105(6):001999. doi: 10.1099/jgv.0.001999 (PMC11261722; doi:10.1099/jgv.0.001999)
Supplement: Uncited Fig. S1. [file jgv-105-01999-s001.pdf]

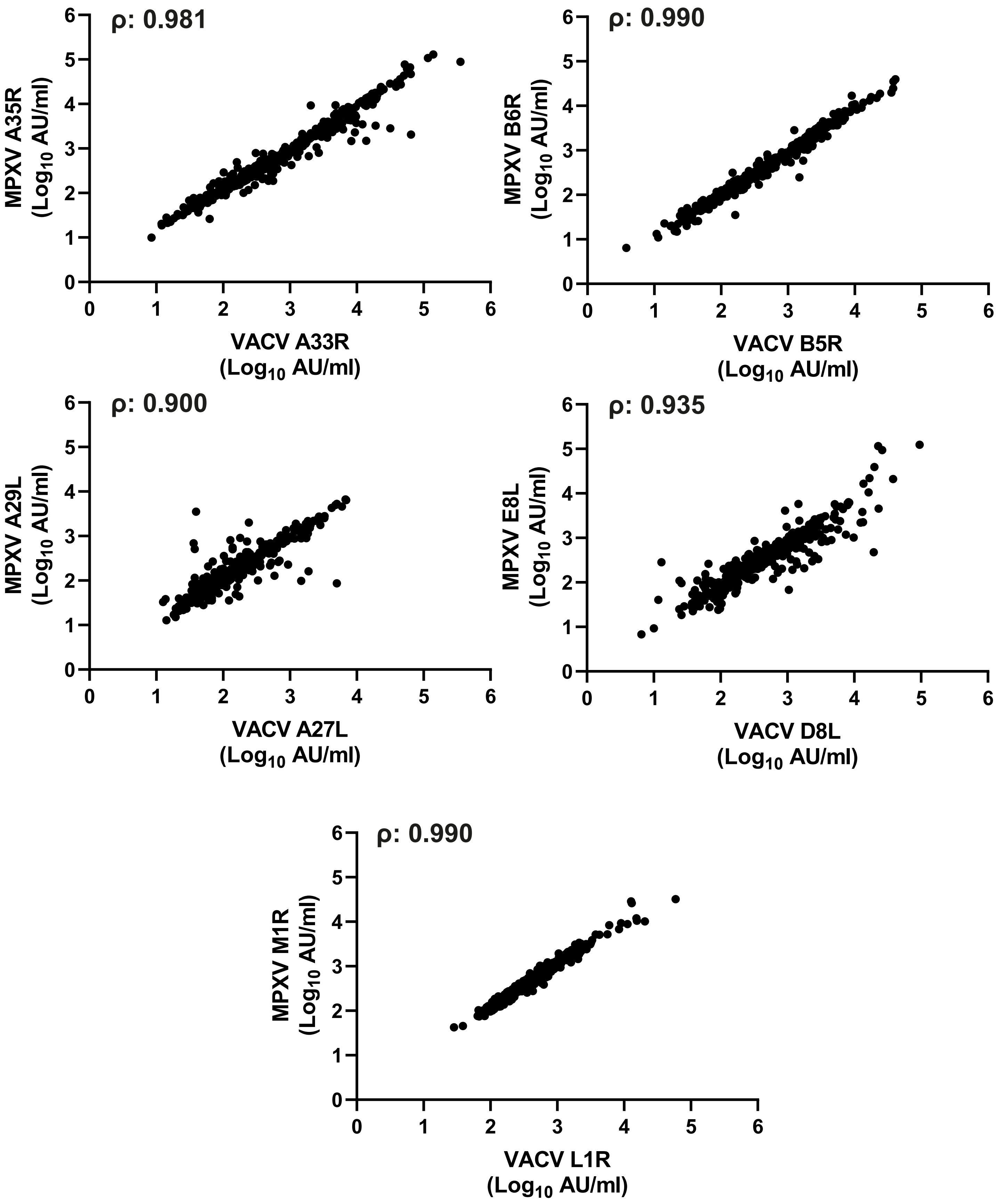

Supplementary figure 1. Correlation between anti-VACV and anti-MPXV binding antibodies. Relative antibody levels against VACV and MPXV epitopes were determined using the Meso Scale Discovery (MSD) Log_{10} arbitrary units (AU). The Log_{10} AU values for the homologous gene products for VACV and MPXV for 430 sera samples were plotted against one another with VACV values on the X-axis and MPXV values on the Y-axis. Spearman's rank correlation test was performed for each epitope pair and the result is expressed in each panel and designated by ρ.

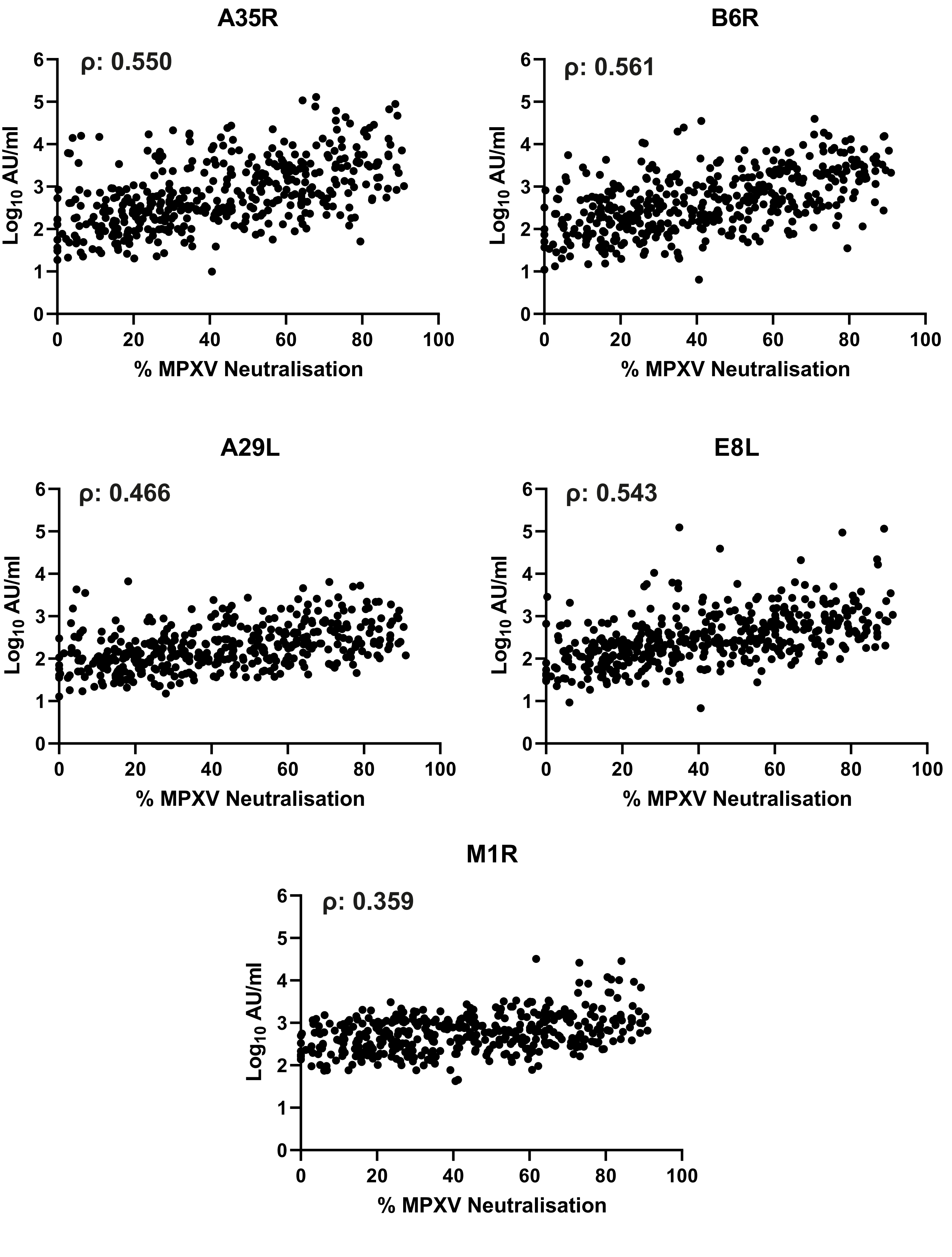

Supplementary figure 2. Correlation between levels of anti-MPXV binding antibodies and MPXV neutralization. The Log10 AU values for 430 sera samples against each of the MPXV epitopes were plotted against the MPXV Neutralization values. Spearman's rank correlation test was performed, and the result is expressed in each panel and designated by ρ.
